# Epidemiological risk factors for adult dengue in Singapore: an 8-year nested test negative case control study

**DOI:** 10.1186/s12879-016-1662-4

**Published:** 2016-07-08

**Authors:** Chee Fu Yung, Siew Pang Chan, Tun Linn Thein, Siaw Ching Chai, Yee Sin Leo

**Affiliations:** Infectious Disease Service, Department of Paediatrics, KK Women’s and Children’s Hospital, Singapore, Singapore; Department of Medicine, Yong Loo Lin School of Medicine, National University of Singapore, Singapore, Singapore; Faculty of Science, Technology and Engineering, La Trobe University, Melbourne, Australia; Communicable Disease Centre, Institute of Infectious Diseases and Epidemiology, Tan Tock Seng Hospital, Singapore, Singapore; Lee Kong Chian School of Medicine, Nanyang Technological University Singapore, Singapore, Singapore; Saw Swee Hock School of Public Health, National University Singapore, Singapore, Singapore

**Keywords:** Dengue, Epidemiology, Test-negative, Risk factors, Public health, Adult, Singapore

## Abstract

**Background:**

Understanding changes in the ecology and epidemiology of dengue is important to ensure resource intensive control programmes are targeted effectively as well as to inform future dengue vaccination strategies.

**Methods:**

We analyzed data from a multicentre longitudinal prospective study of fever in adults using a nested test negative case control approach to identify epidemiological risk factors for dengue disease in Singapore. From April 2005 to February 2013, adult patients presenting with fever within 72 h at selected public primary healthcare clinics and a tertiary hospital in Singapore were recruited. Acute and convalescent blood samples were collected and used to diagnose dengue using both PCR and serology methods. A dengue case was defined as having a positive RT-PCR result for DENV OR evidence of serological conversion between acute and convalescent blood samples. Similarly, controls were chosen from patients in the cohort who tested negative for dengue using the same laboratory methods.

**Results:**

The host epidemiological factors which increased the likelihood of dengue disease amongst adults in Singapore were those aged between 21 and 40 years old (2 fold increase) while in contrast, Malay ethnicity was protective (OR 0.57, 95%CI 0.35 to 0.91) against dengue disease. Spatial factors which increased the odds of acquiring dengue was residing at a foreign workers dormitory or hostel (OR 3.25, 95 % CI 1.84 to 5.73) while individuals living in the North-West region of the country were less likely to get dengue (OR 0.50, 95%CI 0.29 to 0.86). Other factors such as gender, whether one primarily works indoors or outdoors, general dwelling type or floor, the type of transportation one uses to work, travel history, as well as self-reported history of mosquito bite or household dengue/fever were not useful in helping to inform a diagnosis of dengue.

**Conclusions:**

We have demonstrated a test negative study design to better understand the epidemiological risk factors of adult dengue over multiple seasons. We were able to discount other previously speculated factors such as gender, whether one primarily works indoors or outdoors, dwelling floor in a building and the use of public transportation as having no effect on one’s risk of getting dengue.

## Background

Globally, dengue is the most widespread arbovirus that currently infects approximately 390 million people per year [1]. There are four antigenically distinct dengue virus serotypes (DENV-1, DENV-2, DENV-3 and DENV-4). Although the majority of cases resolve uneventfully, in a number of cases, progression to severe dengue or dengue haemorrhagic shock can occur resulting in death. There is no effective treatment for dengue. Currently, the mainstay of dengue prevention strategy relies on surveillance systems to allow early case detection, larval and breeding site source reduction of the main vector *Aedes aegypti* mosquito.

The epidemiology of dengue in Singapore evolved from a childhood disease with significant morbidity and mortality in the 1960s into a period of low dengue incidence between 1974 and 1985 following the introduction of vector source reduction, public health education and law enforcement in the 1970s. Despite continued implementation of these control measures resulting in a national *Aedes spp* House index (HI), which is a measure of the percentage of houses positive for *Aedes spp* breeding, below 1 %, Singapore has experienced a resurgence of dengue since 1986 [2, 3]. The success of vector control activities likely resulted in the reduction of herd immunity in older age groups as well as a fall in force of infection [4]. Dengue became a disease affecting primarily adults with children being spared although serologic surveys have shown that they remain highly susceptible [5]. Previous analysis of case notification data have also found that the prevalence of dengue in women were lower than men as indicated by a male to female disease ratio of 1.6:1 [3, 6, 7] It has been postulated that the reason for such a pattern of dengue epidemiology could be due to a shift in dengue transmission from the home to outside the home [2].

Understanding changes in the ecology and epidemiology of dengue is important for development of more effective control programs [8]. Mosquito control programmes are resource intensive. In Singapore, it was estimated that vector control cost approximately US$50 million per year [9]. Evidence to guide the use and prioritisation of such funds will be invaluable. Dengue being a mosquito-borne disease exhibits demographic as well as spatial and temporal variations in their distribution [10]. However, there is a paucity of long term studies identifying epidemiological risk factors for dengue transmission over multiple years. Understandably most published work describing such risk factors are based on dengue case notification data (clinical and/or laboratory confirmed) from surveillance systems and incidence rate derived from population census [7, 11–13]. Such data and epidemiological analysis have significant limitations. Firstly, it is impossible to exclude selection bias resulting from differences between cases that present to healthcare and those that do not. Secondly, no surveillance system can capture all dengue cases so misclassification of population denominators as non-cases is a constant limitation. Thirdly, the time element is often poorly unaccounted for although dengue epidemiology is season dependent and the force of infection of dengue is known to have temporal variations [14].

The test negative study design has primarily been used and validated to assess vaccine effectiveness [15, 16]. Controls are selected from individuals who also presented to clinicians with similar symptoms to cases and hence were tested for the same disease of interest but found to be negative. In classic case control studies, controls are routinely selected from patients presenting to the same healthcare facility as cases but with a distinct condition from the disease of the cases being studied. For case control results to be valid, controls should belong to the same source population from which cases are identified [17]. They should be individuals who theoretically would have been cases had they acquired the targeted disease or condition of interest. Therefore, test negative controls have significant advantages over classic controls. They provide assurance as being from the same source population as cases would have presented to healthcare and would have been cases if their aetiology had been the disease being studied.

In this paper, we analyzed data from a multicentre longitudinal prospective study of fever in adults using a nested test negative approach to identify epidemiological risk factors for dengue disease over 8 years (2005 to 2013) in Singapore.

## Methods

### Patients

From April 2005 to February 2013, adult patients aged > = 18 years presenting with acute onset fever (> = 37.5 C) or a history of fever (> = 37.5C) within 72 h at selected public primary healthcare clinics and a tertiary hospital in Singapore were recruited. Details of the cohort were published previously [18]. The epidemiological data collected included home address region (as well as dwelling type and floor level if living in multi-storey flat), healthcare recruitment site, travel history in the past 2 weeks, nature of work and primary means of transportation to work (Table 1). There were three scheduled visits: Visit 1 at 1–3 days post fever onset, Visit 2 at 4–7 days post fever onset and visit 3 during convalescence at 3–4 weeks post fever onset. Venous blood was collected at each visit.

**Table 1:** Characteristics of cases and controls

Variable	Category	Case		Control	
		n	%	n	%
Total		395		1308	
Age group	<21	13	3.3	140	10.7
	21 to 30	130	32.9	396	30.3
	31 to 40	97	24.6	238	18.2
	41 to 50	76	19.2	235	18.0
	51 to 60	55	13.9	197	15.1
	61 and above	24	6.1	102	7.8
Gender (Female/Male)	Female	142	35.9	503	38.5
Ethnicity	Chinese	265	67.1	781	59.7
	Malay	33	8.4	240	18.3
	Indian	57	14.4	193	14.8
	Other	40	10.1	94	7.2
Home address region	SW	52	13.4	204	15.8
	C	204	52.4	646	50.0
	NW	62	15.9	264	20.4
	NE	61	15.7	158	12.2
	SE	10	2.6	20	1.5
Country of origin (Singapore/Others)	Singapore	248	62.8	909	69.5
Healthcare recruitment site	A	217	54.9	807	61.7
	B	12	3.0	24	1.8
	C	34	8.6	180	13.8
	D	28	7.1	63	4.8
	E	33	8.4	193	14.8
	F	71	18.0	41	3.1
Medical history (Diabetes/Hypertension/IHD/Malignancy/Steroid treatment) [No/Yes]	No	338	85.6	1099	84.1
Self-reported history of past dengue infection (No/Yes)	No	378	96.7	1235	95.0
Dwelling type	Multistorey public flats	291	73.7	1082	82.8
	Multistorey private flats	18	4.6	64	4.9
	Landed houses	35	8.9	83	6.4
	Foreign (construction) workers dormitory/hostel	51	12.9	77	5.9
Dwelling floor level	mean floor	6	4^a^	7	4^a^
Travel history (No/Yes)	No	342	86.6	1101	84.3
Type of employment (Indoor/Outdoor/Both) and Primary mode of transportation to work (Public-train or bus/Private-taxi or car/Walking)	Unemployed	90	22.8	269	20.7
	Indoor & Public	90	22.8	410	31.6
	Indoor & Private	24	6.1	84	6.5
	Indoor & Walking	10	2.5	41	3.2
	Outdoor & Public	37	9.4	108	8.3
	Outdoor & Private	24	6.1	51	3.9
	Outdoor & Walking	19	4.8	29	2.2
	Both & Public	52	13.2	208	16.0
	Both & Private	35	8.9	80	6.2
	Both & Walking	14	3.5	19	1.5
Self-reported history of mosquito bite (No/Yes)	No	291	73.7	994	76.5
Self-reported history of household dengue (No/Yes)	No	372	94.4	1294	99.2
Self-reported history of household fever (No/Yes)	No	295	74.7	1076	82.6

^a^Standard Deviation

### Laboratory diagnosis

Blood samples were used to diagnose dengue using both PCR and serology methods. RT-PCR was used to detect DENV RNA as previously described [19]. Results were analyzed with the LightCycler software version 3.5. Reactions with high crossover point (Cp) or ambiguous melting curve results were further analyzed by 2 % agarose gel electrophoresis, to confirm the presence of the correctly sized amplicon. Serology testing for IgM and IgG antibodies against DENV was performed using commercially available ELISAs (Panbio, Brisbane, Australia) according to manufacturer’s instructions.

### Definitions

To ensure high sensitivity and specificity thus minimizing bias from misclassification, cases and controls were classified using strict laboratory criteria. A dengue case was defined as having a positive RT-PCR result for DENV OR evidence of serological conversion between acute and convalescent blood samples. Similarly, controls were chosen from patients in the cohort whom we could be confident that they did not have dengue. This was defined as having a negative RT-PCR result for DENV OR no evidence of serological conversion between acute and convalescent blood samples.

### Statistical analysis

Variables percentage proportions between cases and controls were calculated. Data with continuous variables were compared using two tailed *t*-test. Multilevel regression analysis incorporating time by month and year was used for multivariable analysis. First we developed a full model of dengue which included all independent variables: predetermined age categories, gender, ethnicity, home address region, country of birth, healthcare recruitment site, past medical history, self-reported history of past dengue infection, dwelling type, dwelling floor level, travel history, nature of work, transportation to work, self-reported history of mosquito bite, self-reported history of household dengue and self-reported history of household fever. Backward stepwise regression was performed to identify independent predictor variables of dengue disease. We fixed age and gender but other variables were only retained in the model if *P* < 0.05. All statistical analysis was performed using STATA, version 13. We estimated that a sample size of 395 cases and 10 % exposed controls would allow us to detect highest odds ratio (OR) < 1 of 0.4 and smallest OR > 1 of 2, with α at 0.05 and β of 0.1.

## Results

**Fig. 1:**
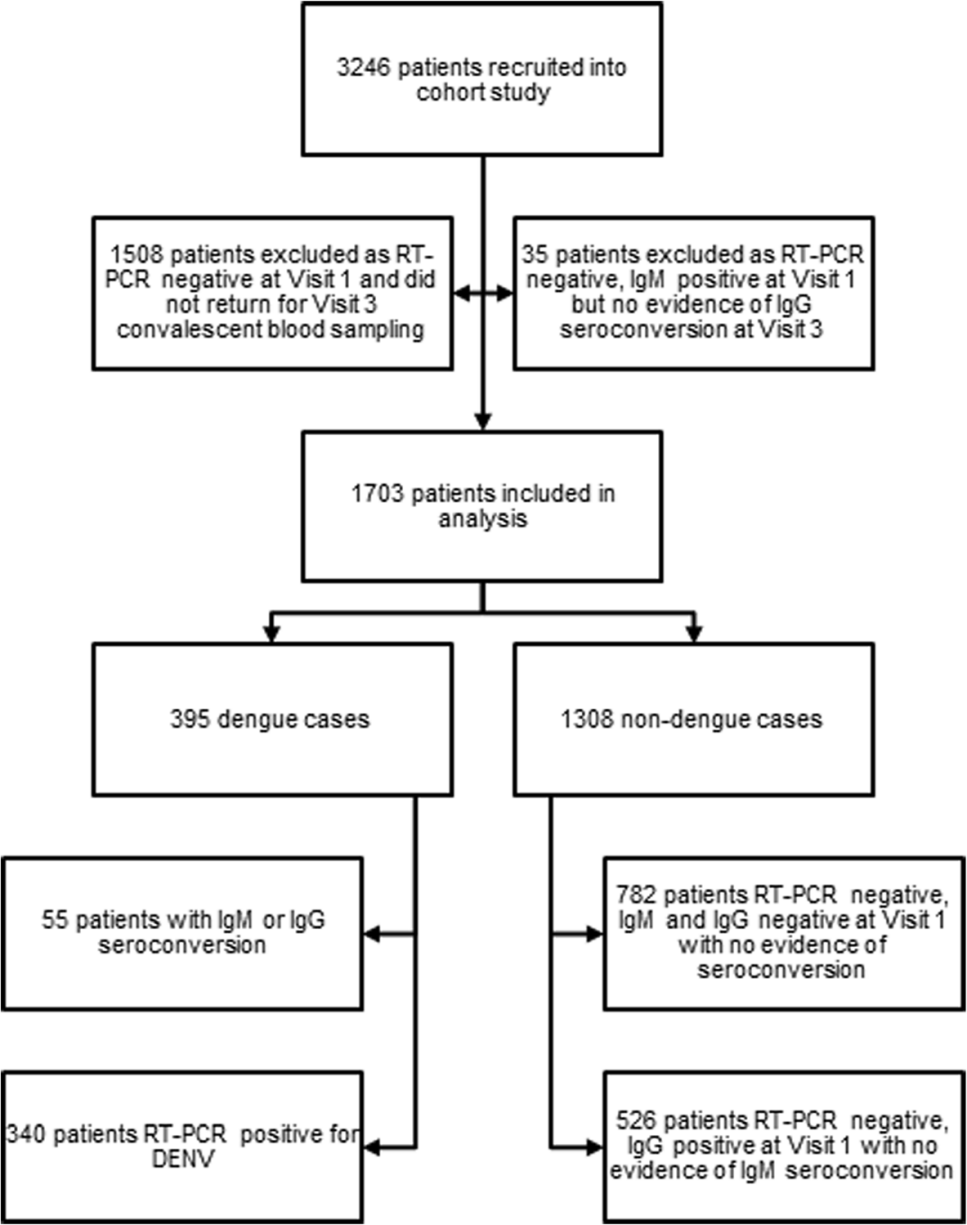
Consort diagram of cases and controls used in the study

**Fig. 2:**
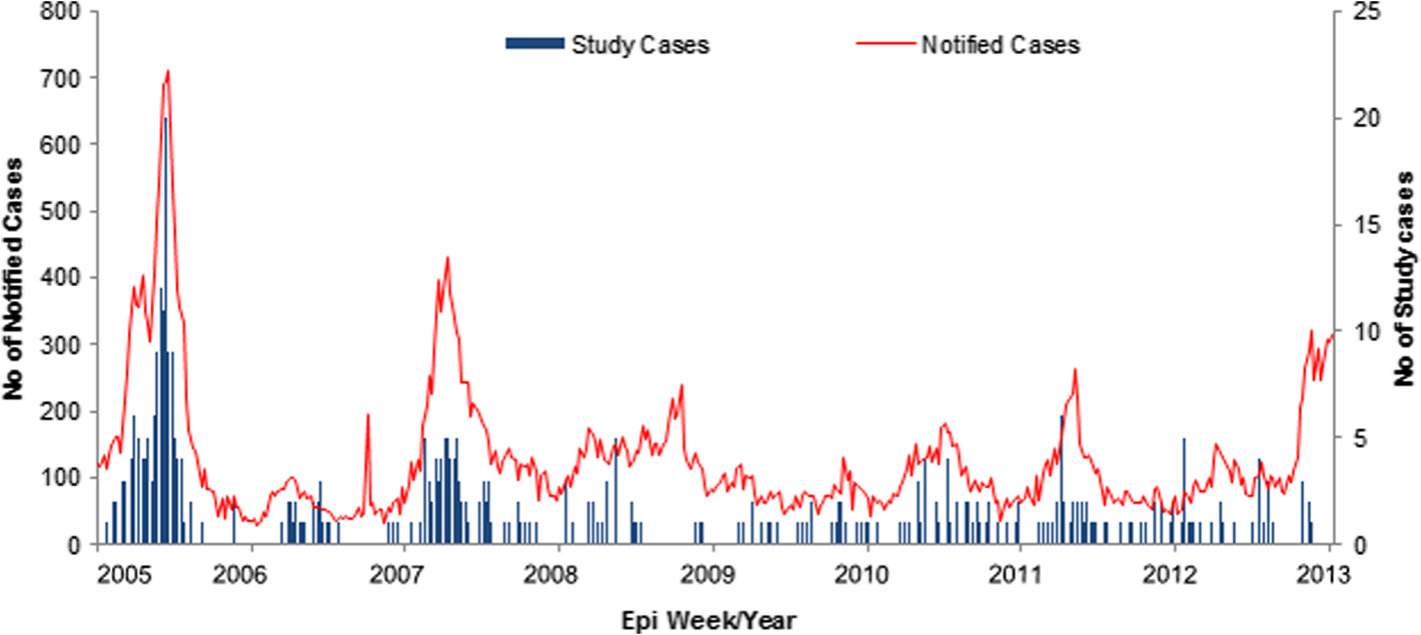
Distribution of dengue study cases and national notification cases, 2005 to 2013

A total of 3246 patients were recruited for the prospective cohort study. 1508 were excluded because they were RT-PCR negative and also did not return for the convalescent visit 3. Another 35 patients were also excluded as they were RT-PCR negative and did not demonstrate IgG seroconversion despite being IgM positive at Visit 1. The remaining 1703 patients fulfilled our case definitions for cases and controls and were used for subsequent analysis (Fig. 1). Summary demographics and epidemiological characteristics between the study cases and controls are shown in Table 1. Overall, cases and controls were fairly similar. The temporal distribution of study dengue cases also resembled the national notification of dengue cases as shown in Fig. 2.

In the full model where all variables were included, statistically significant odds ratios for dengue disease were found for housing type and healthcare recruitment site variables only (Table 2). However, after backward stepwise analysis where age and gender were fixed, only ethnicity, home address region and housing type remained as relevant variables in the model. Individuals aged between 21 and 30 years (OR 2.12, 95 % CI 1.11 to 4.05) and 21 to 40 years (OR 2.28, 95 % CI 1.16 to 4.47) were found to have significantly higher odds of dengue disease compared to those < 21 years. The point estimate OR in individuals aged > 41 years showed a downward trend signifying lower risk of dengue disease although the 95 % CI were not statistically significant. There was no difference in odds of dengue by gender. Geographically, apart from residents in the North West region of Singapore who had a lower risk of dengue (OR 0.50, 95 % CI 0.29 to 0.86), the rest of the island had similar risk. Interestingly, Malay ethnicity was found to be protective against dengue disease with an OR 0.57, 95 % CI 0.35 to 0.91 when compared to Chinese ethnicity. We also found that individuals residing at foreign worker dormitory/hostels were more likely to be diagnosed with having dengue compared to those residing in multi-storey public flats (OR 3.25, 95 % CI 1.84 to 5.73).

**Table 2:** Multilevel logistic regression analysis of dengue epidemiological risk factors

Variable	Category	Full model		Stepwise model	
		OR	95 % CI	AOR	95%CI
Gender	Female	1.00		1.00	
	Male	1.05	0.72 to 1.53	0.95	0.70 to 1.31
Age Group	< 21	1.00		1.00	
	21 to 30	**2.56**	**1.24 to 5.29**	**2.12**	**1.11 to 4.05**
	31 to 40	**3.01**	**1.39 to 6.53**	**2.28**	**1.16 to 4.47**
	41 to 50	2.53	1.17 to 5.47	1.83	0.92 to 3.62
	51 to 60	2.35	1.04 to 5.34	1.62	0.79 to 3.31
	61 and above	1.68	0.63 to 4.51	1.18	0.51 to 2.72
Ethnicity	Chinese	1.00		1.00	
	Malay	**0.47**	**0.28 to 0.79**	**0.57**	**0.35 to 0.91**
	Indian	0.69	0.43 to 1.12	0.73	0.47 to 1.14
	Other	0.63	0.33 to 1.22	0.82	0.47 to 1.44
Home address region	South-west	1.00		1.00	
	Central	0.63	0.28 to 1.39	0.65	0.42 to 1.02
	North-west	**0.38**	**0.15 to 0.98**	**0.50**	**0.29 to 0.86**
	North-east	0.74	0.32 to 1.77	1.20	0.71 to 2.03
	South-east	1.21	0.36 to 4.12	1.25	0.44 to 3.54
Housing type	Multistorey public flats	1.00		1.00	
	Multistorey private flats	0.80	0.37 to 1.73	0.73	0.35 to 1.52
	Landed houses	0.99	0.28 to 3.55	1.44	0.82 to 2.55
	Foreign workers dormitory/hostel	**3.03**	**1.51 to 6.06**	**3.25**	**1.84 to 5.73**
Country of origin	Singapore	1.00			
	Others	0.92	0.61 to 1.38		
Healthcare recruitment site	A	1.00			
	B	**4.27**	**1.15 to 15.95**		
	C	0.65	0.24 to 1.74		
	D	**3.60**	**1.52 to 8.54**		
	E	1.61	0.69 to 3.76		
	F	**2.38**	**1.08 to 5.26**		
Self-reported history of past dengue infection	No	1.00			
	Yes	0.63	0.28 to 1.41		
Medical history (Diabetes/Hypertension/HD/Malignancy/Steroid treatment)	No	1.00			
	Yes	0.58	0.34 to 0.99		
Dwelling floor level	Level 1	1.00			
	Every 1 level up	0.95	0.91 to 0.99		
Travel history	No	1.00			
	yes	0.87	0.55 to 1.37		
Type of employment (Indoor/Outdoor/Both) and Primary mode of transportation to work (Public-train or bus/Private-taxi or car/Walking)	Unemployed	1.00			
	Indoor & Public	0.51	0.31 to 0.82		
	Indoor & Private	0.87	0.42 to 1.80		
	Indoor & Walking	0.70	0.28 to 1.78		
	Outdoor & Public	0.70	0.35 to 1.41		
	Outdoor & Private	1.41	0.63 to 3.11		
	Outdoor & Walking	0.68	0.24 to 1.91		
	Both & Public	0.68	0.38 to 1.21		
	Both & Private	0.43	0.19 to 0.98		
	Both & Walking	0.81	0.23 to 2.88		
Self-reported history of mosquito bite	No	1.00			
	Yes	0.91	0.62 to 1.35		
Self-reported history of household dengue	No	1.00			
	Yes	1.02	0.23 to 4.52		
Self-reported history of household fever	No	1.00			
	Yes	1.22	0.83 to 1.80		

Figures in **bold** are statistically significant, *P* < 0.05

*OR* Odds Ratio, *AOR* Adjusted Odds Ratio, *CI* Confidence Interval

## Discussion

The host epidemiological factors which increase the likelihood of dengue disease amongst adults in Singapore were found to be age, particularly if one was aged between 21 and 40 years old while in contrast, Malay ethnicity was found to lower the odds of dengue disease. Spatial epidemiological factors which increased the risk of dengue was residing at a foreign workers dormitory or hostel while individuals living in the North West region of the country had a lower risk of dengue. We also confirmed that there were no differences in odds of dengue disease by gender. Other factors such as whether one primarily works indoors or outdoors, unemployed status, dwelling type or floor, the type of transportation one uses to work, travel history, as well as self-reported history of mosquito bite or household dengue/fever were not useful in helping to inform a diagnosis of dengue. We have demonstrated a test negative control study design incorporating temporal adjustment using multi-level analysis to better understand the epidemiological risk factors of dengue over multiple seasons.

Our results defined a clear and specific age group with increased risk of dengue over the past 8 years in Singapore. Individuals aged 21 to 40 years old have the highest risk of acquiring the disease amongst adults. This is likely to be the age group with the highest risk for the whole population as serologic studies have confirmed very low rates of infection in children compared to adults [5, 20]. Furthermore, the point odds ratios of dengue in age categories above 40 years clearly showed a downward trend. This has important implications for future roll out of a dengue vaccine. The leading dengue vaccine candidate is a recombinant live, attenuated, tetravalent dengue vaccine (Sanofi Pasteur) which demonstrated overall efficacy of 56 · 5 % [21]. However, these results were based on trial cohorts composed of children in settings with high paediatric dengue incidences. Strategically, one would normally aim to deploy a vaccine at an age before the highest risk of infection or disease. Our epidemiological study show that if a dengue vaccine were deployed during the first year of life, in a setting with older age of first infection such as Singapore, the benefits of the vaccine would not be detectable until 20 years later. Currently, we do not know if protection will persist that long. A rise in median age of infection reported in developing countries like Thailand, Vietnam and anecdotally across Asia suggests that Singapore’s epidemiology may not be unique in the near future [12, 22–25]. Furthermore, older age of dengue disease was also commonly reported in non-endemic subtropical regions in Taiwan and China who have large seasonal outbreaks following importation of the virus [13, 26]. Therefore, a pre-school leaving or even an adult dengue vaccination strategy may need to be considered as a more rational and cost effective approach for these settings. The challenge is there is unlikely to be efficacy data for adults in the near future to help inform policy decisions. Hence any roll out would require close monitoring to assess efficacy as well as safety. In addition, adult vaccination strategies are renowned to be logistically very difficult to implement and achieve high coverage. This has been demonstrated in countries where adult influenza and pneumococcal vaccination programmes have been rolled out. For a vector borne disease like dengue, high coverage to reduce the reproductive number to < 1 should be an important target to ensure herd protection in the population with the ultimate objective of disease elimination.

Surveillance data routinely reports higher number of dengue cases and clusters in the eastern and central regions of Singapore [27]. Our analysis suggests that this is probably an artefact of higher population densities in these regions than actual higher dengue transmission. We postulate that our finding of lower risk of dengue in the North West region compared to the rest of the island could also be attributed to the difference in level of urbanization with subsequent variations in mosquito vector species. The North West region of Singapore is significantly less developed than the rest of the island with large areas of forests still present. This would count against the main vector of dengue, the *Aedes Aegypti* species which is known to thrive in urban settings rather than forested areas [28–31]. Unfortunately, we do not have vector surveillance data to verify this and cannot rule out other factors influencing spatial dengue risk. We did find the odds of dengue disease to be more than 3 times higher in individuals living at foreign workers dormitories or hostels compared to multi-storey public flats where the majority of the local population resides. Such settings could be high-risk areas for dengue epidemics. Dengue transmission has been shown to vary at the neighbourhood level within an urban setting in Thailand [32]. Therefore, the densely occupied nature of such dormitories or hostels in combination with low herd immunity since a significant proportion of foreign workers come from countries where dengue is not endemic may be the reason for this. However, the workers mostly work at construction sites and these have been known to be high risk areas by authorities with mosquito breeding found in about 10 % of sites inspected in 2013 [33]. Singapore has introduced tough legislation for guilty contractors including stop-work orders, prosecution in court and requirements to employ environmental control officer at large construction sites. It is difficult to disentangle the actual site of dengue infection for this cohort although individuals with dengue during the highly infectious fever phase are more likely to be resting at home. Strategies including both educational and source reduction activities targeted at occupants as well as owners of foreign worker residential sites may need to be considered as part of public health efforts to control dengue.

We were not able to identify any difference in odds of dengue disease by factors such as gender, working outdoors, being unemployed and type or level of dwelling. Previous studies have suggested that dengue transmission in Singapore have shifted to outside the home environment based on a jump in seroprevalence from 1 % in < 6 years old to about 7 % in 6 to 15 years which is the age for formal schooling in Singapore [5]. Similarly, it was also hypothesized that lower reported females incidence could be explained by the same dynamics when factoring differences in male and female proportions in the workforce [6]. However, the pediatric seroprevalence data did not have participants older than 15 years and participants were recruited from a single hospital setting. Large seroprevalence studies carried out in adults > 18 years old have shown that > 5 % increases in seroprevalence for 5 to 10 year age categories should be expected [20, 34]. Gender differences in health seeking behaviour and employment rates where producing a medical certificate is a requirement if one is unwell in Singapore could have accounted for dengue gender variations found in routine notification data. Since our controls were individuals who also presented to healthcare but tested negative for dengue, our results were not vulnerable to these biases. Furthermore, an adult serologic survey using samples from a representative National Health Survey also did not find that those who spent more time at home e.g. homemaker or retired as being more likely to be positive for recent dengue infection [34]. Outbreak reports using notification data from the two large outbreaks (2005 and 2007) covered by the study period had conflicting reports of increased risk of dengue by dwelling types [7]. The scarcity of landed properties in Singapore makes landed properties an important socioeconomic profile which may affect health service seeking behaviour and access. Our study design using robust controls confirmed that the type and floor level one resides in does not influence one’s risk of dengue disease. This lends support to the homogenous success of national vector control activities and the known significant vertical flight range of the *Aedes* mosquito [35].

Clinically, we were able to confirm that self-reported history of mosquito bite or household dengue/fever and travel history elicited during consultations were not useful in predicting a diagnosis of dengue amongst febrile patients, confirming the known challenges in clinical dengue diagnosis. Interestingly, we did identify Malay ethnicity as being almost 40 % less likely to have dengue compared to Chinese ethnicity amongst adults presenting with fever. Serologic surveys support this finding, with Malay ethnicity identified as the ethnic group with the lowest seropositivity [20, 34]. A genetic predisposition to dengue fever or severe dengue is suspected and studies in Vietnam and Cuba have found a possible role for HLA class 1 allele [36, 37]. A recent study in neighbouring Malaysia which has a similar ethnic mix to Singapore identified HLA-B*13 and HLA-B*18 as being associated with dengue susceptibility and protection respectively amongst patients with Malay ethnicity.

In this study we were not able to account for the role of dengue infection. We used febrile adults presenting to healthcare with a confirmed diagnosis of dengue as a proxy for dengue transmission. However, surveillance notification data are also subject to the same limitation. Although serologic surveys can identify dengue infection especially seroprevalence, available tests are limited by their ability to identify actual date of infection. We also assumed that our study cohort was representative of dengue epidemiology in Singapore. Comparing our incidence to national notification data covering the same period showed similar distribution which would support our assumption (Fig. 2). We were not able to investigate serotype specific differences which may be present in view of known variations in viral competence. DENV-1 and DENV-2 were the predominant circulating serotypes during the study period.

## Conclusions

Our results provided invaluable insights into deciphering the epidemiology of adult dengue in urban settings. We demonstrated the use of test negative controls which minimized biases associated with notification data routinely used for studying dengue epidemiological risk factors. In Singapore, the age group most likely to acquire dengue disease was found to be between 21 to 40 years but Malay ethnicity was found to be protective against dengue. We also identified living in a foreign worker dormitory/hostel as a significant epidemiological risk factor for dengue disease. Spatially, the risk of dengue was significantly lower in the North West region of Singapore compared to the rest of the island possibly due to its lower level of urbanization resulting in reduced *Aedes Aegypti* prevalence. We confirmed that gender, employment, use of public transportation, dwelling type as well as floor did not affect one’s odds of getting dengue. Our findings have important implications in informing clinical diagnosis of adult dengue, prioritising vector control activities as well as future roll out of a licensed dengue vaccine.
